# Long-term analysis to objectify the tumour grading by means of automated microscopic image analysis of the nucleolar organizer regions (AgNORs) in the case of breast carcinoma

**DOI:** 10.1186/1746-1596-8-56

**Published:** 2013-04-08

**Authors:** Klaus-Jürgen Winzer, Joachim Bellach, Peter Hufnagl

**Affiliations:** 1 Clinic for Gynaecology – Breast Center, Berlin, Germany; 2 Comprehensive Cancer Center Charité, Berlin, Germany; 3 Institute of Pathology, Charité - Universitätsmedizin Berlin, Charité-Platz 1, Berlin, 10117, Germany

**Keywords:** Breast cancer, Grading, Image analysis, AgNOR quantification, Survival analysis, Cox regression

## Abstract

**Background:**

Apart from a number of cases of inaccurate prognosis in regard to individual patients, the inter- and intra-observer variability of the classical, histological prognosis parameters have been under repeated discussion. For this reason, a long-term analysis was carried out in regard to overall survival by means of automated microscopic image analysis of the nucleolar organizer regions (AgNORs) to objectify tumour grading in the case of breast carcinoma.

This consists of a selective representation of argyrophilic proteins that are associated with the nucleolus organising regions.

**Methods:**

The evaluation included 244 female patients with an average age of 59.3 years. The characterisation of the histological sections was carried out on the basis of the AMBA/R system. With this software the histometric characterisation level was evaluated in terms of the nucleolus organizer regions. The post-observation data were obtained from the clinical register and were complemented by mortality data from the cancer registers and by data supplied by the residents’ registration office of Berlin.

**Results:**

The average post-observation period was 106.6 months. With the Cox-Regression the influence of the co-variables (conventional prognosis parameters and AgNOR parameters) were examined. In the model, only the parameters pN, G and various AgNOR parameters remain present.

**Conclusion:**

There is a strong correlation between survival and selected AgNOR parameters. These could replace the conventional grading as the standard measure for the mitosis rate together with the pleomorphism level. Instead of the-time consuming AMBA/R system originally used, a new implementation of AgNOR quantification with modern VM systems could be applied.

**Virtual slides:**

The virtual slide(s) for this article can be found here: http://www.diagnosticpathology.diagnomx.eu/vs/1449591192859058.

## Introduction

The adjuvant (and neo-adjuvant) systemic therapy reduces the clinically significant metastasis according to subtype of the primary non-metastasised breast carcinoma to differing proportions. The patient expects the attending physician to weigh the benefits (better tumour-specific survival) against the so-called costs (worsened quality of life caused by the therapy). In the comparison between tumour-specific survival and overall survival, however, are to be included, even to a minor degree, cases of treatment-delayed mortality (through cardiotoxic drugs or the development of solid secondary tumours or acute forms of leukaemia), which must be added to the costs. For the evaluation of the individual risk of distant metastasis, the established prognosis factors, size of the tumour, lymph node status, grading, PVI (peritumoral venous invasion) and the estrogen receptor and progesteron receptor status as well as the HER-2/new-overexpression were taken into account. This risk-adapted selection for adjuvant therapy is reflected in the St. Gallen conferences on breast carcinoma. Additionally, in 2009 [1], in this regard, the proliferation index (Ki67 labelling index and/or the histopathological description of the mitosis index) and also gene signatures were discussed.

The determination of the proteins uPA (urokinase-type plasminogen activator) and its counterpart PAI-1 (bio-marker PAI-1 and uPA [2,3]), particularly propagated in Germany, was unable to assert itself internationally. Instead, gene signatures on the basis of “micro array analysis” or the “RT-PCR method” are becoming increasing established commercially as Oncotype DX [4] or “MammaPrint” [5]. In German-speaking pathology, to ensure independence from the aforesaid commercial suppliers, the Endopredict-Test [6] has been developed. Additionally, by way of genome analysis using DNA micro arrays, subtypes have been defined [7], the prognostic significance of which was finally highlighted at the 2011 St. Gallen Conference [8].

A number of other research areas concerning prognosis and histological differentiation of breast carcinoma will only be mentioned briefly. Apart from the identification of appropriate gene signatures, a model of human breast cancer progression has been in development for a number of years. While researchers until recently assumed a linear path from flat epithelial atypia (FEA) via atypical ductal hyperplasia (ADH) and DCIS to invasive ductal carcinoma, new findings using transcriptomic and epigenetic technologies are pointing to at least one further molecular genetic pathway [see [9] for a summary of molecular based publications]. A number of review articles on a multitude of prognostic paraters including AgNOR have been published [see [10] for a summary] without yet entering the therapy recommendations of the relevant consensus conferences. Nevertheless, women carrying a germline mutation on the BRCA-1 or BRCA-2 gene are accepted into an early recognition program reflecting a risk for not only earlier but more aggressive forms breast cancer [11]. The β1 integrin expression is not only correlated to survival, but as a cell adhesion molecule represent a prognosis factor for metastasis that is independent of cell proliferation [12]. Mammaglobin-A and –B are parameters specific to breast carcinoma [13].

Besides the individual imprecision in the prognosis with regard to individual patients, there has been repeated discussion on the inter- and intra-observer variability of the classical, histological prognosis parameters. Moreover, the standardisation of the immune histochemical results is criticised because it involves a semi-quantitative procedure. The results for HER-2/new are given as approximately 20% inaccurate [14]. Also other studies criticised this imprecision [15] and attempted to establish alternative methods of diagnosis [16].

For this reason, we took the opportunity to conduct a long-term analysis in regard to overall survival by means of automated microscopic image analysis of the nucleolar organizer regions (AgNORs) to objectify the tumour grading in the case of breast carcinoma.

This involves a selective depiction of argyrophilic proteins that are associated with the nucleolus organizer regions. The quantity of the AgNOR particles and/or their surface parameters correlate with the proliferation activity and hence could constitute a standardised parameter for the tumour grading as prognosis marker.

Research via PubMed (1 February 2012), revealed only 114 hits for the combination of “AgNOR” and “breast cancer” during the years from 1989 to 2011. In the Charité, since the mid-1980s, various “AgNOR” working groups engaged themselves with a variety of different tumour entities also including, for example, breast cancer and its preliminary stages [17](overview)[18-21]. Originally, the AMBA (automatic microscopic image analysis) of the AgNOR promised to obtain more precisely defined information in relation to survival compared with the conventional method of grading in the case of breast cancer.

Therefore, only those AgNOR parameters that in earlier evaluations showed an univariate, significant correlation with overall survival were included in the analysis. The individual AgNOR parameters are described in detail elsewhere [22,23].

## Material and method

The evaluation included 244 female patients with an average age of 59.3 years (24–92) with the parameters recorded in Table 1. Of these, 43.4% received a breast-conserving therapy (2.9% without and 40.5% with lymphadenectomy) as well as 56.6% a mastectomy (2.9% without and 53.7% with lymphadenectomy). Whereby 72.5% of the female patients included in this study were hormone receptor positive. The histological samples originate from female patients who received operative treatment in the surgical clinic of the Charité (now called Campus Mitte) in the years 1989–1997. The selection of the women patients was mainly determined by the existing residual material in the form of paraffin blocks at the Pathological Institute of the Charité. From the original 267 measurements on samples with an invasive mamma carcinoma, 10 women patients with a different malignant tumour and 13 patients with a contralateral mamma carcinoma prior to the period of data registration of the breast examined here, respectively, were excluded.

**Table 1:** Evaluated parameters

**Parameters**	**Definition**	**n**
**Conventional parameters**		
pT	Size of the invasive tumour components	244
pN	Lymph node involvement	235
G	Tumour grading	242
**AgNOR parameters**		
nornbc_m	Mean value of the corrected number of the AgNORs per cell nucleus (the AgNORs lying very close together in the cell nucleus are recorded as a conglomerate and counted accordingly)	242
nor_k1	mean number (percentage) of nuclei contained in 1 AgNOR	244
nor_k2	mean number (percentage) of nuclei contained in 2 AgNORs	244
nor_k4	mean number (percentage) of nuclei contained in 4 AgNORs	244
nor_k5	mean number (percentage) of nuclei contained in 5 AgNORs	244
nor_k6	mean number (percentage) of nuclei contained in 6 AgNORs	244
nor_k7	mean number (percentage) of nuclei contained in 7 AgNORs	244
nor_k8	mean number (percentage) of nuclei contained in 8 AgNORs	244
snar_r_v	Standard deviations of the surface of the single AgNOR in relation to the total surface of the AgNORs per cell nucleus in μm^2^ per mille	244
center_v	Standard deviation of the position of the AgNORs located in the centre of the cell nucleus	244
cent_r_v	Standard deviation of proportion of AgNORs in a central position	244
bord_r_v	Standard deviation of the proportion of the AgNORs located in the defined, peripheral zone in the cell nucleus	244
locat_v	Position of the NORs	244
mnrat2_m	Mean ratio between largest AgNOR and total AgNOR area size per cell nucleus in per mille	244

The characterisation of the histological sections was carried out on the basis of the AMBA/R system. With this software, instead of using the karyometric characterisation level, as was usual before, the histometric characterisation level was used [24]. The detailed description including illustrations of the AgNOR measurement and the AgNOR featores was published in [25].

The post-observation data were obtained through consultations with the patients in the special out-patient surgery of the university. Since this did not include all results (mortalities), these were complemented by mortality data from the combined cancer register of the new Federal States and Berlin as well as by data from the residents’ registration office of Berlin. This method led to an overestimation of the cases of death in the Kaplan-Meier survival graph [26]. Hence, the mortality rates are not comparable, but this kind of data collection led to an increase of the events.

## Results

The average post-observation of the 244 women patients was 106.6 (0 – 247) months.

First, the results of the Cox Regression (in reverse, step-by-step) are shown for the parameters in Table 1 in regard to overall survival (Tables 2 and 3) and breast cancer specific survival (Tables 4 and 5).

**Table 2:** Evaluation of the case processing in respect of overall survival

**Case classes**		**N**	**Percent**
Cases available for analysis	Event^a^	81	33.2%
	Censored	150	61.5%
	Total	231	94.7%
Unused cases	Cases with missing values	12	4.9%
	Censored cases prior to earliest event in one layer	1	.4%
	Total	13	5.3%
Total		244	100.0%

a. Dependent variable: post-observation (months) with additional data from the cancer register and residents’ registration office.

**Table 3:** Remaining parameters of the Cox Regression in reverse step-by-step (Likelihood Ratio) after Step 11 in regard to overall survival

**Parameters**	**Definition**	**Significance**
pN	Lymph node involvement	.001
G	Tumour grading	.021
nornbc_m	Mean value of the corrected number of the AgNORs per cell nucleus	.036
nor_k8	Mean number (percentage) of nuclei contained in 8 AgNORs	.097
cent_r_v	Standard deviation of the proportion of AgNORs in a central position	.022
locat_v	Position of the NORs	.081
mnrat2_m	Mean ratio between largest AgNOR and total AgNOR area size per cell nucleus in per mille	.021

**Table 4:** Evaluation of the case processing in respect of breast cancer specific survival

**Case classes**		**N**	**Percent**
Cases available for analysis	Results^a^	46	18.9%
	Censored	185	75.8%
	Total	231	94.7%
Unused cases	Cases with missing values	12	4.9%
	Censored cases prior to earliest event in one layer	1	.4%
	Total	13	5.3%
**Total**		**244**	**100.0%**

a. Dependent variable: post-observation (months) with additional data from the cancer register and residents’ registration office.

**Table 5:** Remaining parameters of the Cox Regression in reverse step-by-step (Likelihood Ratio) after Step 11 in regard to breast cancer specific survival

**Parameters**	**Definition**	**Significance**
pT	Size of tumour	.026
pN	Lymph node involvement	.000
G	Tumour grading	.056
nor_k1	mean number of nuclei contained in 8 AgNORs	.107
cent_r_v	Standard deviation of the proportion of AgNORs in a central position	.047
locat_v	Position of the NOR´s	.011
mnrat2_m	Mean ratio between largest AgNOR and total AgNOR area size per cell nucleus in per mille	.036

Combining the AgNOR parameters that were not significant for survival in the Cox model into a few parameters using factor analysis did not produce different results.

Factor analysis of the AgNOR parameters did not yield additional insights. The AgNOR parameters with the higher levels of significance had appropriate weights in the generated factors.

Excluding the five AgNOR parameters remaining in Table 3 from the factor analysis before regressing again did not yield any new significant factors.

**Figure 1:**
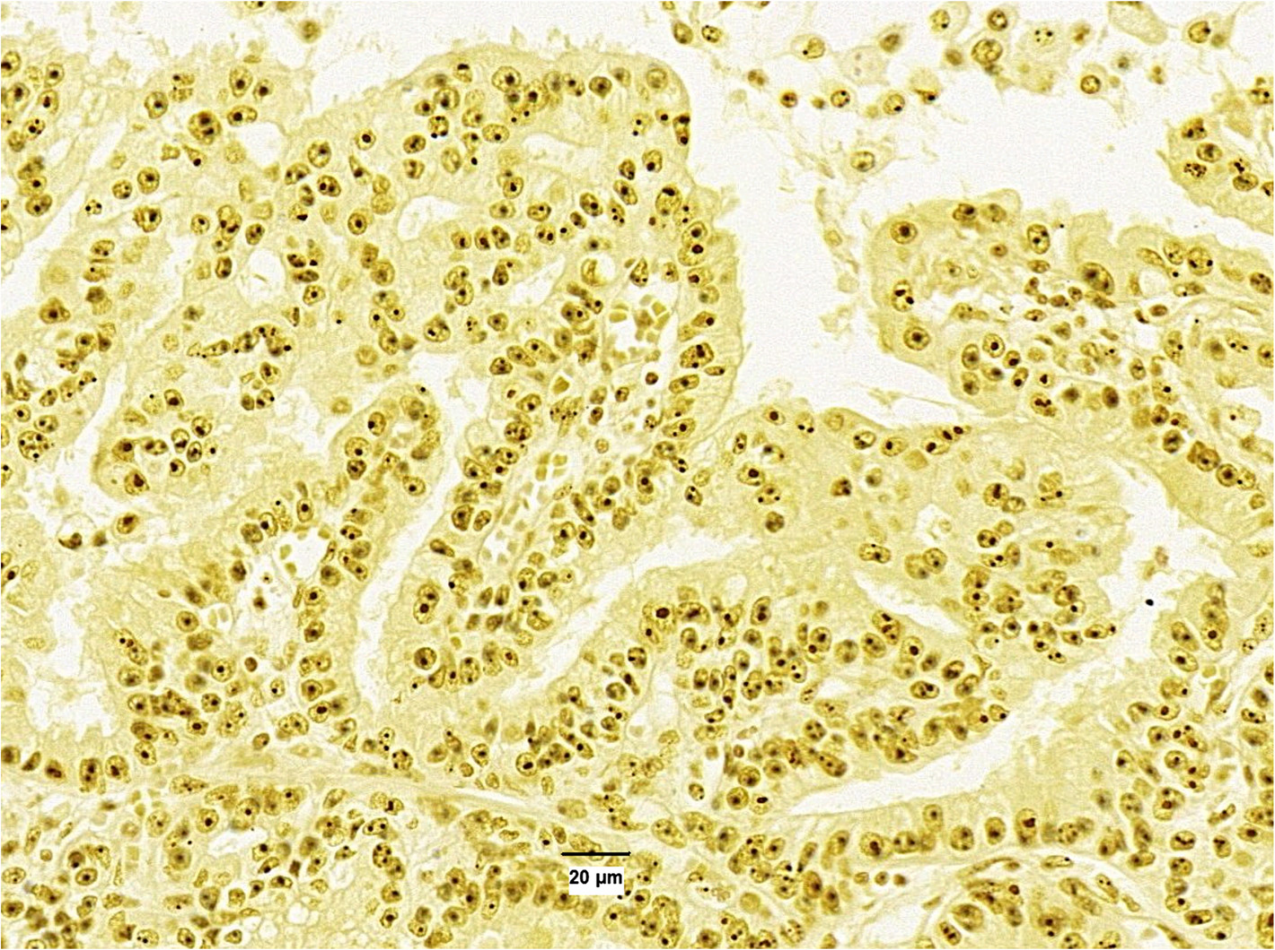
Low variability of size, number and location of AgNORS in cell nucleus.

**Figure 2:**
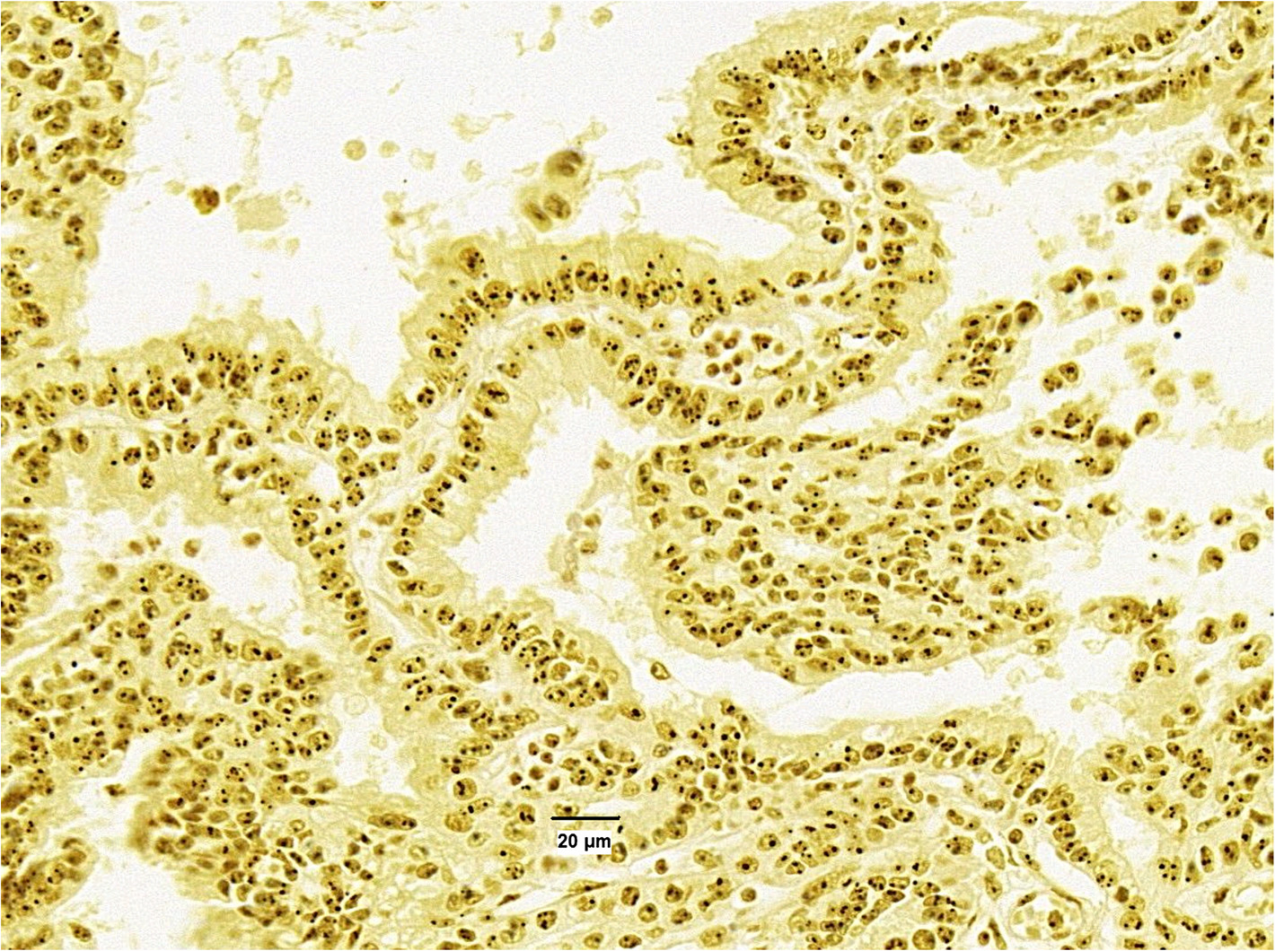
High variability in number, size and location of AgNORs in cell nuclei.

To generate possible further studies, new sub-groups have been formed from the existing file. In this is included, in addition to the usual grading, a seven-stage grading (corresponding to the number of points according to Bloom and Richardson in the modification according to Elston and Ellis [27] and the polymorphism as well as the AgNOR parameter “position of the NORs” (locat_v) and the mean ratio between largest AgNOR and total AgNOR area size per cell nucleus in per mille (mnrat2_m). This is illustrated in Figures 1 and 2. The evaluation is again carried out for overall survival (Tables 6 and 7) and breast cancer specific survival (Tables 8 and 9).

**Table 6:** Evaluation of the case processing in respect of overall survival

**Case classes**		**N**	**Per cent**
Cases available for analysis	Results^a^	46	18.9%
	Censored	88	36.1%
	Total	134	54.9%
Unused cases	Cases with missing values	110	45.1%
**Total**		**244**	**100.0%**

a. Dependent variable: post-observation (months) with additional data from the cancer register and residents' registration office.

**Table 7:** Cox Regression in reverse step-by-step (Likelihood Ratio) in regard to overall survival

**Variables in the equation**		**Significance**
Step 1	g	.860
	pkt_grad	.636
	polym	.035
	mnrat2_m	.262
	locat_v	.639
Step 2	pkt_grad	.490
	polym	.035
	mnrat2_m	.225
	locat_v	.656
Step 3	pkt_grad	.463
	polym	.039
	mnrat2_m	.062
Step 4	polym	.001
	mnrat2_m	.045

**Table 8:** Evaluation of the case processing in respect of breast cancer specific survival

**Case classes**		**N**	**Per cent**
Cases available for analysis	Results^a^	28	11.5%
	Censored	106	43.4%
	Total	134	54.9%
Unused cases	Cases with missing values	110	45.1%
	Total	110	45.1%
Total		244	100.0%

a. Dependent variable: post-observation (months) with additional data from the cancer register and residents’ registration office.

**Table 9:** Cox Regression in reverse step-by-step (Likelihood Ratio) in regard to breast cancer specific survival

**Step and feature**		**Significance**
Step 1	g	.695
	pkt_grad	.398
	polym	.045
	mnrat2_m	.854
	locat_v	.217
Step 2	g	.717
	pkt_grad	.410
	polym	.045
	locat_v	.179
Step 3	pkt_grad	.289
	polym	.047
	locat_v	.178
Step 4	polym	.001
	locat_v	.132
Step 5	polym	.002

## Discussion

The evaluation was carried out for overall survival and breast cancer specific survival, respectively, since, on the one hand, long-term overall survival includes therapy-related mortality, as already described in detail in the introduction, and, on the other hand, the cause of death statistics based on the death certificates is regarded as imprecise. To obtain more results, the mortality value is over-rated due to the way in which the data was collected, as analysed in detail elsewhere [26].

The Cox Regression showed that various AgNOR parameters, both in relation to overall survival as well as breast cancer specific survival, possess additional information compared with the routinely determined parameters pT, pN and G.

Other authors describe that the number of AgNORs correlates with tumour-free survival [28] or that the AgNOR analysis represents an additional tool for identifying, in the case of a limit-value HER2 status, further patients that can be considered for a trastuzumab therapy [29]. The AgNOR protein quantity is also said to correlate with the survival rate [30]. There were descriptions of a positive correlation between the AgNOR score and the histological grading [31,32] and/or the number of the AgNORs in relation to the degree of degeneration [33]. It is explained that this correlation is due to the circumstance that the ribosomal biogenesis can be quantified by the morphometric analysis of AgNORs [34]. The growth rate or proliferation activity of a tumour cell depends on the proportion of proliferating cells (growing cell fraction), shown as a percentage of the MIB-1 fraction, and on the cell division rate. The cell division rate correlates with the AgNOR parameters [35]. This could also explain the tendency towards a lower correlation of the mitosis rate or the absolute number of mitoses per 10 brightness fields in relation to the selected AgNOR parameters compared with the pleomorphism. Due to the small number of cases of these parameters, which emerged from routine data, however, there were no significant correlations. Therefore, this is not shown separately in the results section.

The majority of the citations made here by other working groups represent the results of recording the AgNOR of cytological samples in the case of a flow cytophotometry or of the recorded percentage of a defined area of a histological slide.

In the case of breast cancer, it is not a matter of a homogeneous accumulation of tumour cells as, for example, in the case of urinary bladder cancer, but rather involves up to 7 different cell types located in the area of the tumour. Hence, in the application of AMBA/R, a semi-automatic method was used, whereby the cells to be included in the evaluation were initially marked manually. This perhaps explains the somewhat better correlation.

## Conclusions

There is a strong correlation between survival and selected AgNOR parameters. These could replace the conventional grading as the standard measure for the mitosis rate together with the pleomorphism level. This, however, is so far only a hypothesis generated by the study, which would have to be confirmed by an investigation with a larger number of cases. However, with the AMBA/R system applied here, the relatively high time consumption is disadvantageous. In view of the development in the field of virtual microscopy (VM), one should consider a new implementation of AgNOR quantification. In modern VM systems it is possible to analyse many more cell nuclei in a much shorter time.

## Consent

Written informed consent was obtained from the patient for publication of scientific results and accompanying histological images based on paraffin blocks.

## Competing interests

There are no competing interests of the authors.

## Authors’ contributions

All clinical parts have been performed by K-JW. This includes general study design, diagnostics, patient management and data collection. Image analysis and AgNOR quantification was done by PH. JB was responsible for the statistical analysis. All authors read and approved the final manuscript.

## References

[B1] Goldhirsch A Ingle JN Gelber RD Coates AS Thürlimann B Senn HJ Panel members Thresholds for therapies: highlights of the St Gallen International Expert Consensus on the primary therapy of early breast cancer 2009 Ann Oncol 2009 20 1319 1329 19535820 10.1093/annonc/mdp322 PMC2720818

[B2] Schmitt M Goretzki L Jänicke F Calvete J Eulitz M Kobayashi H Chucholowski N Graeff H Biological and clinical relevance of the urokinase-type plasminogen activator (uPA) in breast cancer Biomed Biochim Acta 1991 50 731 741 1801751

[B3] Schmitt M Harbeck N Brünner N Jänicke F Meisner C Mühlenweg B Jansen H Dorn J Nitz U Kantelhardt EJ Thomssen C Cancer therapy trials employing level-of-evidence-1 disease forecast cancer biomarkers uPA and its inhibitor PAI-1 Expert Rev Mol Diagn 2011 11 617 634 21745015 10.1586/erm.11.47

[B4] Mamounas EP Tang G Fisher B Paik S Shak S Costantino JP Watson D Geyer CE Jr Wickerham DL Wolmark N Association between the 21-gene recurrence score assay and risk of locoregional recurrence in node-negative, estrogen receptor–positive breast cancer: results from NSABP B-14 and NSABP B-20 J Clin Oncol 2010 28 1677 1683 20065188 10.1200/JCO.2009.23.7610 PMC2849763

[B5] van't Veer LJ Paik S Hayes DF Gene expression profiling of breast cancer: a new tumor marker J Clin Oncol 2005 23 1631 1635 15755970 10.1200/JCO.2005.12.005

[B6] Filipits M Rudas M Jakesz R Dubsky P Fitzal F Singer CF Dietze O Greil R Jelen A Sevelda P Freibauer C Müller V Jänicke F Schmidt M Kölbl H Rody A Kaufmann M Schroth W Brauch H Schwab M Fritz P Weber KE Feder IS Hennig G Kronenwett R Gehrmann M Gnant M EP Investigators A new molecular predictor of distant recurrence in ER-positive, HER2-negative breast cancer adds independent information to conventional clinical risk factors Clin Cancer Res 2011 17 6012 6020 21807638 10.1158/1078-0432.CCR-11-0926

[B7] Perou CM Sørlie T Eisen MB van de Rijn M Jeffrey SS Rees CA Pollack JR Ross DT Johnsen H Akslen LA Fluge O Pergamenschikov A Williams C Zhu SX Lønning PE Børresen-Dale AL Brown PO Botstein D Molecular portraits of human breast tumours Nature 2000 406 747 752 10963602 10.1038/35021093

[B8] Goldhirsch A Wood WC Coates AS Gelber RD Thürlimann B Senn HJ Panel members Strategies for subtypes--dealing with the diversity of breast cancer: highlights of the St. Gallen International Expert Consensus on the Primary Therapy of Early Breast Cancer 2011 Ann Oncol 2011 22 1736 1747 21709140 10.1093/annonc/mdr304 PMC3144634

[B9] Bombonati A Sgroi DC The molecular pathology of breast cancer progression J Pathol 2011 223 307 317 21125683 10.1002/path.2808 PMC3069504

[B10] Elzagheid A Kuopio T Pyrhönen S Collan Y Lymph node status as a guide to selection of available prognostic markers in breast cancer: the clinical practice of the future? Diagn Pathol 2006 1 41 17092354 10.1186/1746-1596-1-41 PMC1654187

[B11] Zhang Q Zhang Q Cong H Zhang X The ectopic expression of BRCA1 is associated with genesis, progression, and prognosis of breast cancer in young patients Diagn Pathol 2012 7 181 23276146 10.1186/1746-1596-7-181 PMC3541118

[B12] dos Santos PB Zanetti JS Ribeiro-Silva A Beltrão EI Beta 1 integrin predicts survival in breast cancer: a clinicopathological and immunohistochemical study Diagn Pathol 2012 7 104 22894137 10.1186/1746-1596-7-104 PMC3523034

[B13] MariadelasMercedes N Fernando P Florencia P Néstor L Hugo K Silvana N Alejandro G Alejandra A Boris E Denninghoff VC Miembro de la Carrera de Investigador del Consejo Nacional de Investigaciones Científicas y Técnicas (CONICET) Immunohistochemical characterization of neoplastic cells of breast origin Diagn Pathol 2012 7 73 22726568 10.1186/1746-1596-7-73 PMC3468373

[B14] Wolff AC Hammond ME Schwartz JN Hagerty KL Allred DC Cote RJ Dowsett M Fitzgibbons PL Hanna WM Langer A McShane LM Paik S Pegram MD Perez EA Press MF Rhodes A Sturgeon C Taube SE Tubbs R Vance GH van de Vijver M Wheeler TM Hayes DF American Society of Clinical Oncology; College of American Pathologists American Society of Clinical Oncology/College of American Pathologists Guideline Recommendations for Human Epidermal Growth Factor Receptor 2 Testing in Breast Cancer J Clin Oncol 2007 25 118 145 17159189 10.1200/JCO.2006.09.2775

[B15] Noske A Loibl S Darb-Esfahani S Roller M Kronenwett R Müller BM Steffen J von Toerne C Wirtz R Baumann I Hoffmann G Heinrich G Grasshoff ST Ulmer HU Denkert C von Minckwitz G Comparison of different approaches for assessment of HER2 expression on protein and mRNA level: prediction of chemotherapy response in the neoadjuvant GeparTrio trial (NCT00544765) Breast Cancer Res Treat 2011 126 109 117 21190079 10.1007/s10549-010-1316-y

[B16] Hiroaki N Kelly BD Padilla M Nikolaus W Brunhoeber P Bai I Singh S Ranger-Moore J Bieniarz C Hitoshi T Grogan TM A gene-protein assay for human epidermal growth factor receptor 2 (HER2): brightfield tricolor visualization of HER2 protein, the HER2 gene, and chromosome 17 centromere (CEN17) in formalin-fixed, paraffin-embedded breast cancer tissue sections Diagn Pathol 2012 7 60 22647525 10.1186/1746-1596-7-60 PMC3487810

[B17] Guski H Hufnagl P Freitag A Winzer K-J Automatisierte Mikroskopbildanalyse und Prognose von Präneoplasien und Karzinomen der Brustdrüse Gegenbaur's morphol Jahrb 1989 135 39 53 2544475

[B18] Guski H Winzer K-J Hufnagl P Wolf G Reichert S Automated grading in breast cancer by image analysis of histological sections Acta Stereol 1990 9 259 270

[B19] Guski H Winzer K-J Seidenfaden U Hufnagl P Wolf G Häufigkeitsverteilung, mikroskopische Bildanalyse und Grading des Mammakarzinoms Zentralbl Pathol 1991 137 249 255 1718410

[B20] Guski H Hufnagl P Kaufmann O Krause K Winzer KJ AgNOR Analysis of atypical ductal hyperplasia and intraductal carcinoma of the breast Anal Quant Cytol Histol 2000 22 206 212 10872036

[B21] Günther L Hufnagl P Winzer K-J Guski H Different proliferation patterns in breast cancer: AgNOR measurements in ER-negative and ER-positive tumor cells Anal Cell Pathol 2000 20 155 162 11205318 10.1155/2000/914765 PMC4617517

[B22] Hufnagl P Guski H Schulz HJ Measuring of AgNORs using image analysis Zentralbl Pathol 1994 140 31 35 7515670

[B23] Günther L Hufnagl P Technique and feasibility of a dual staining method for estrogen receptors and AgNORs Anal Cell Pathol 2000 20 151 154 11205317 10.1155/2000/565976 PMC4618433

[B24] Hufnagl P Wenzelides K Methoden der Charakterisierung histologischer Schnittpräparate auf der Basis des AMBA/R-Systems Gegenbaurs Morphol Jahrb 1989 135 33 38 2661302

[B25] Hufnagl P Beil M Wenzelides K Martin H Die Vermessung der Anzahl, Größe und Lage von AgNORs in histologischen Schnitten von Astrozytomen Zentralbl Pathol 1991 137 493 497 1805927

[B26] Winzer K-J Bellach J Wertigkeit der routinemäßig erfassten Nachsorgedaten bei Brustkrebspatientinnen Zentbl Chir 2010 153 257 261 10.1055/s-0030-1247381 20549588

[B27] Elston CW Ellis IO Pathological prognostic factors in breast cancer. I. The value of histological grade in breast cancer: experience from a large study with long-term follow-up Histopathology 1991 19 403 410 1757079 10.1111/j.1365-2559.1991.tb00229.x

[B28] Abboud P Lorenzato M Joly D Quereux C Birembaut P Ploton D Prognostic value of a proliferation index including MIB1 and argyrophilic nucleolar organizer regions proteins in node-negative breast cancer Am J Obstet Gynecol 2008 199 1 7 18455135 10.1016/j.ajog.2008.02.025

[B29] Bánkfalvi A Giuffrè G Ofner D Diallo R Poremba C Buchwalow IB Barresi V Böcker W Tuccari G Relationship between HER2 status and proliferation rate in breast cancer assessed by immunohistochemistry, fluorescence in situ hybridisation and standardised AgNOR analysis Int J Oncol 2003 23 1285 1292 14532967

[B30] Ceccarelli C Trerè D Santini D Taffurelli M Chieco P Derenzini M AgNORs in breast tumours Micron 2000 31 143 149 10588060 10.1016/s0968-4328(99)00071-2

[B31] Kesari AL Chellam VG Nair PP Madhavan J Nair P Nair MK Pillai MR Tumor proliferative fraction in infiltrating duct carcinoma Gen Diagn Pathol 1997 143 219 224 9489954

[B32] Sinha SK Singh UR Bhatia A Gupta S Cytomorphological features, AgNOR counts and c-erb B-2 in carcinoma breast J Indian Med Assoc 1998 96 71 76 9828547

[B33] Krüger S Fahrenkrog T Müller H Proliferative and apoptotic activity in lobular breast carcinoma Int J Mol Med 1999 4 171 174 10402484 10.3892/ijmm.4.2.171

[B34] Treré D Ceccarelli C Montanaro L Tosti E Derenzini M Nucleolar size and activity are related to pRb and p53 status in human breast cancer. Anonymous J Histochem Cytochem 2004 52 1601 1607 15557214 10.1369/jhc.4A6454.2004

[B35] Treré D Ceccarelli C Migaldi M Santini D Taffurelli M Tosti E Chieco P Derenzini M Cell proliferation in breast cancer is a major determinant of clinical outcome in node-positive but not in node-negative patients Appl Immunohistochem Mol Morphol 2006 14 314 323 16932023 10.1097/00129039-200609000-00010

